# Thromboembolic complication in Essential Thrombocythemia

**Published:** 2012-11-19

**Authors:** Zahra Mozaheb

**Affiliations:** 1 Department of Hematology, Imam Reza Hospital, Mashhad university of Medical Science, Iran

**Keywords:** Thrombocythemia, microvascular occusions, gangrene

## Image in Medicine

**Figure 1:**
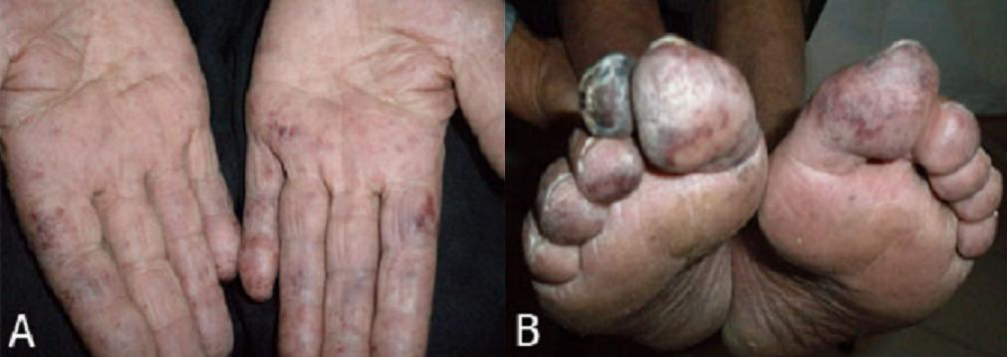
Symmetrical reddinenig and mottling of toes and fingers (A), and toe gangrene (B)

The presenting symptoms of patients with essential thrombocythemia are quite variable. After detection of thrombocytosis about 13 to 37 percent of patients relate symptoms due to hemorrhagic event, and about 22 to 84 percent of patients report thromboembolic complication. The thrombotic events primarily involved the microvasculature, with thrombosis of large vessels occuring far less frequently. Microvascular occusions involving the toes and finger are frequent. In this report we introduce a patient with paresthesia and peripheral gangrene. A 62-year old man was admited with headach, burning pain and erythromelalgia in feet and hands associated with symmetrical reddinenig and mottling of toes and fingers (Figure 1-A). In physical examination the patient was not severely ill at diagnosis, and the karnofsky score was 85, and splenomegaly was not detectable. Patient also had gangrene in forth toe of the rigth feet (Figure 1-B). Laboratory findings of patient included: platelet counts: 1,600,000/µl, leukocyte count: 18000/µl, hemoglobin level: 15.2 gr/dl. In peripheral blood smear there was severe platelet aggregation and platelet anisocytosis with megathrombocytes, and variouse morphologic abnormality in size and shape of red cells. Bone marrow aspiration findings were, increased marrow cellularity, marked megakaryocytic hyperplasia, with morphologically bizarre megakaryocytes, nuclear pleomorphism and clustering of megakaryocytes. The diagnosis of essential thrombocytemia was established by being posetive of JAK2 mutation.

